# Ultra-low-dose vs. standard-of-care-dose CT of the chest in patients with post-COVID-19 conditions—a prospective intra-patient multi-reader study

**DOI:** 10.1007/s00330-024-10754-z

**Published:** 2024-05-09

**Authors:** Christian Wassipaul, Daria Kifjak, Ruxandra-Iulia Milos, Florian Prayer, Sebastian Roehrich, Melanie Winter, Lucian Beer, Martin L. Watzenboeck, Svitlana Pochepnia, Michael Weber, Dietmar Tamandl, Peter Homolka, Wolfgang Birkfellner, Helmut Ringl, Helmut Prosch, Benedikt H. Heidinger

**Affiliations:** 1 Department of Biomedical Imaging and Image-guided Therapy, Medical University of Vienna, Vienna, Austria; 2 Department of Radiology, UMass Memorial Medical Center and University of Massachusetts Chan Medical School, Worcester, MA USA; 3 Imaging Verbund, Vienna, Austria; 4 contextflow GmbH, Vienna, Austria; 5 Center for Medical Physics and Biomedical Engineering, Medical University of Vienna, Vienna, Austria; 6 Department of Diagnostic and Interventional Radiology, Clinic Donaustadt, Vienna Healthcare Group, Vienna, Austria

**Keywords:** Computed tomography, Ultra-low-dose CT, Lung, Post-COVID, Diagnostic accuracy

## Abstract

**Objectives:**

To conduct an intrapatient comparison of ultra-low-dose computed tomography (ULDCT) and standard-of-care-dose CT (SDCT) of the chest in terms of the diagnostic accuracy of ULDCT and intrareader agreement in patients with post-COVID conditions.

**Methods:**

We prospectively included 153 consecutive patients with post-COVID-19 conditions. All participants received an SDCT and an additional ULDCT scan of the chest. SDCTs were performed with standard imaging parameters and ULDCTs at a fixed tube voltage of 100 kVp (with tin filtration), 50 ref. mAs (dose modulation active), and iterative reconstruction algorithm level 5 of 5. All CT scans were separately evaluated by four radiologists for the presence of lung changes and their consistency with post-COVID lung abnormalities. Radiation dose parameters and the sensitivity, specificity, and accuracy of ULDCT were calculated.

**Results:**

Of the 153 included patients (mean age 47.4 ± 15.3 years; 48.4% women), 45 (29.4%) showed post-COVID lung abnormalities. In those 45 patients, the most frequently detected CT patterns were ground-glass opacities (100.0%), reticulations (43.5%), and parenchymal bands (37.0%). The accuracy, sensitivity, and specificity of ULDCT compared to SDCT for the detection of post-COVID lung abnormalities were 92.6, 87.2, and 94.9%, respectively. The median total dose length product (DLP) of ULDCTs was less than one-tenth of the radiation dose of our SDCTs (12.6 mGy*cm [9.9; 15.5] vs. 132.1 mGy*cm [103.9; 160.2]; *p* < 0.001).

**Conclusion:**

ULDCT of the chest offers high accuracy in the detection of post-COVID lung abnormalities compared to an SDCT scan at less than one-tenth the radiation dose, corresponding to only twice the dose of a standard chest radiograph in two views.

**Clinical relevance statement:**

Ultra-low-dose CT of the chest may provide a favorable, radiation-saving alternative to standard-dose CT in the long-term follow-up of the large patient cohort of post-COVID-19 patients.

## Key Points

- *CT is the modality of choice for follow-up of patients with post-COVID-19 conditions but is associated with radiation exposure.*
- *Ultra-low-dose CT of the chest offers excellent intrareader agreement and high accuracy compared to standard-dose CT in this population.*
- *Ultra-low-dose CT of the chest provides a radiation-saving alternative to standard-dose CT in the follow-up of post-COVID patients.*

## Introduction

Since the beginning of the ongoing coronavirus disease 2019 (COVID-19) pandemic, more than 770 million cases have been officially registered worldwide [1]. Although symptoms gradually improve in most patients, some individuals show persistent symptoms for more than four weeks, or even new symptoms, termed by the Centers for Disease Control and Prevention (CDC) as “Long COVID” or “post-COVID-19 conditions” [2–7]. The CDC estimates that 15.2% of all adults in the USA have suffered from post-COVID-19 conditions, while a large Dutch survey showed that 12.7% of COVID-19 patients report symptoms for more than three months [8, 9]. To evaluate those post-COVID patients with persistent symptoms of morphological lung abnormalities, such as fibrotic-like changes, they are recommended to undergo computed tomography (CT) imaging evaluation [10–12]. Indeed, depending on the severity of COVID-19, follow-up CT scans at three to six months after the diagnosis show residual lung changes ranging from 18% in mild outpatient cases to 78% in formerly hospitalized patients and an average prevalence of approximately 30% at follow-up within one year [13–18].

However, CT examinations are inherently linked to radiation exposure with lifetime dose and an associated cancer risk accumulating with repeated examinations [19]. Improvements in third-generation CT scanners with novel reconstruction algorithms, filtering techniques, and detectors have resulted in the potential for considerable reductions in radiation dose by up to 95% for various indications of chest CTs, termed ultra-low-dose CT (ULDCT) [20–24]. The performance of ULDCT has been previously investigated for the detection of acute COVID-19 pneumonia in the context of the ongoing pandemic [25–27], as well as for the evaluation of specific lung abnormalities, such as pulmonary nodules, pulmonary emphysema, and pneumonia [22, 28–30]. No evidence, however, exists on the performance of ULDCT in patients with post-COVID conditions and its agreement with standard-of-care-dose CT (SDCT) in this cohort consisting of millions of patients who are vulnerable to accumulating substantial lifetime radiation exposure.

Therefore, the aim of this study was to conduct an intrapatient comparison of ULDCT and SDCT of the chest in terms of the diagnostic accuracy of ULDCT and intrareader agreement in patients with post-COVID conditions.

## Materials and methods

This prospective study was approved by the local institutional review board (No. 2254/2018), and written, informed consent was obtained from all participants.

### Study population

In this study, all consecutive patients referred to our department for a chest CT for the follow-up of COVID-19 between May 2020 and January 2021 were screened for inclusion and exclusion criteria.

Inclusion criteria were: (a) clinical chest CT indication for follow-up of prior COVID-19; (b) history of COVID-19 confirmed by a positive PCR / antigen test, by typical symptoms with a consecutive positive antibody test, or typical symptoms of COVID-19 along with a COVID-19-positive contact person; (c) more than 4 weeks between the diagnosis of COVID-19 and the CT exam; (d) persistent symptoms at the time of referral, consistent with post-COVID-19 conditions; (e) 18 years or older; and (f) ability to provide informed consent. There were no exclusion criteria due to the strictly clinical indication for the CT examination and the very low radiation dose added by the ULDCT scan.

### Image acquisition

All patients consecutively received our institutional SDCT and an additional ULDCT scan of the chest within the same examination, based on the same scan projection radiograph (scout view) and with the same scan range. Only examinations from a single point in time were regarded for each patient. All CT scans were acquired on the same third-generation 2 × 128-row multidetector dual-source CT System (Somatom Drive, Siemens Healthineers) in helical scan mode, and the patient in a supine body position, with arms elevated and in full inspiratory breath-hold.

CT acquisition for SDCT scans was performed at 110 ref. kVp with automatic tube voltage selection and a tube current of 81 ref. mAs with tube current modulation enabled (CAREDose in setting “normal,” Siemens Healthineers). For ULDCT scans, the targeted, effective radiation dose was 0.2 mSv, corresponding to twice the mean effective radiation dose of a standard CXR examination in two views (0.1 mSv) [31–33]. Therefore, the parameters for ULDCT scans were based on an earlier investigation and tailored to acquire scans at a dose length product (DLP) of approximately 12.5 mGy*cm for a standard-sized patient, corresponding to an effective dose (ED) of 0.22 mSv [34]. Accordingly, the ULDCT scan protocol comprises a fixed tube voltage of 100 kVp with tin filtration and a tube current of 50 ref. mAs with tube current modulation enabled (in setting “weak”), with all other scan parameters identical to the SDCT protocol.

Image reconstruction for SDCT scans was done using a lung kernel (I70f) and a soft tissue kernel (I31f) in transverse and coronal orientations, at a section thickness (ST) of 3 mm with a reconstruction interval (RI) of 2 mm, and an ST of 1 mm with an RI of 0.8 mm. An iterative reconstruction algorithm at level 3 of 5 (ADMIRE, Siemens Healthineers) was used for all SDCT images. ULDCT scans were reconstructed by applying lung kernel I50f at iteration level 5 of 5, with all other parameters identical to SDCT reconstructions. CT acquisition and reconstruction parameters are detailed in Table 1.

**Table 1:** CT acquisition and reconstruction parameters

Siemens Somatom Drive	SDCT	ULDCT
Tube voltage	110 kVp automatic tube voltage selection	100 kVp fixed
Tin filtration	disabled	enabled
Tube current time product	81 ref. mAs	50 ref. mAs
Tube current modulation CAREDose	enabled (normal)	enabled (weak)
Collimation	128 × 0.6 mm	128 × 0.6 mm
Gantry rotation time	0.5 s	0.5 s
Pitch	1.2	1.2
Lung kernel	I70f, ADMIRE level 3 of 5	I50f, ADMIRE level 5 of 5
Soft tissue kernel	I31f, ADMIRE level 3 of 5	I31f, ADMIRE level 5 of 5
Slice thickness / reconstruction interval	1 / 0.8 mm (lung kernel) 3 / 2 mm (soft tissue kernel) 12 / 3 mm (MIPs)	1 / 0.8 mm (lung kernel) 3 / 2 mm (soft tissue kernel) 12 / 3 mm (MIPs)

### CT assessment

All SDCT and ULDCT scans were independently evaluated by all four readers (two expert chest radiologists—H.P. and I.R.M., with 20 and 12 years of experience at the time of reading, respectively; two residents in radiology—F.P. and B.H.H., two residents in their third year of training, respectively), who were blinded to all imaging reports and clinical data. The two expert chest radiologists, as well as the two resident radiologists, were randomly assigned to one of two study arms, respectively. Readers assigned to arm A (one expert, one resident) first evaluated all SDCTs and then the ULDCTs, while those assigned to arm B (one expert, one resident) first evaluated the ULDCTs and then the SDCTs. For each reader, the evaluations of the two dose levels were at least 3 weeks apart, and patients were presented in separate random orders to minimize recall bias.

CT images were assessed with the local Picture Archiving and Communication System software (IMPAX EE R20 XIX, Agfa Healthcare) and were presented to the readers in standard lung window settings with the option to freely adjust level and width for improved visualization.

For every CT scan, readers first assessed subjective image quality on a five-point Likert scale with ‘1’ referring to non-diagnostic and ‘5’ to optimal image quality [35–37]. Second, they evaluated the scans for the presence of the following lung abnormalities as defined by the Fleischner glossary of terms [38]: ground glass opacities (GGO); consolidations; reticulations; honeycombing; parenchymal bands; crazy paving; lung nodules; bronchial wall thickening; bronchial enlargement; and pleural effusion. Third, lung abnormalities were assessed with regard to their consistency with post-COVID-19 lung abnormalities [10, 39]. In cases in which the readers determined that these findings were consistent with post-COVID lung abnormalities, they were asked to estimate the total extent of affected lung parenchyma as a percentage of total lung volume. Additionally, readers reported the affected lobes along with the transverse, sagittal, and coronal distribution.

Volume Computed Tomography Dose Index (CTDIvol, mGy), as well as the Dose Length Product (DLP, mGy*cm), were extracted from CT imaging protocol data. To allow for intermodal comparison, the ED was estimated by multiplying the DLP by the conservative conversion factor for chest CTs of 0.017 (mSv*mGy^−1^*cm^−1^) [40].

### Statistical analysis

Statistical analyses were performed using SPSS Statistics version 27.0 (IBM) by a biostatistician with more than 25 years of experience (M.W.). Continuous variables are expressed as mean ± standard deviation or as median [interquartile range], as appropriate. Categorical variables are expressed as absolute numbers and their percentages.

The reference standard was built based on the majority decision (at least three of the four readers) from the SDCT scans, and, in case of a tie, the most experienced expert reader in lung imaging, H.P., served as a tiebreaker.

Intra-reader agreement of ULDCT with SDCT with respect to the specific lung abnormalities was calculated by comparing ULDCT with the reference standard and tested for statistical significance using a McNemar test without multiplicity to prevent an increase in type two error. With respect to the extent of lung involvement, intrareader agreement was computed using a multilevel analysis with an unstructured covariance matrix, considering multiple measurements as well as accounting for missing values if a reader did not detect post-COVID lung abnormalities in the respective patient. Findings were evaluated with regard to the intrareader agreement between ULDCT and SDCT. Additionally, the diagnostic performance of ULDCT was computed for patients with an examination more than 12 weeks after the onset of COVID-19 in order to account for more stringent definitions of post-COVID-19 conditions [7, 41, 42] and were assessed for statistical significance by using generalized estimating equation models to take into account multiple measures per patient.

Inter-reader agreement for the dichotomous presence of post-COVID lung abnormalities and specific imaging findings was calculated via the Fleiss’ Kappa [43]. The intraclass correlation coefficient (ICC) was applied for the assessment of the interreader agreement regarding the extent of affected lung parenchyma (two-way random for absolute agreement).

The subjective image quality of the two radiation dose levels was analyzed using a Wilcoxon signed ranks test. *P* values equal to or below 0.05 were considered statistically significant.

## Results

### Patient recruitment and characteristics

**Fig. 1:**
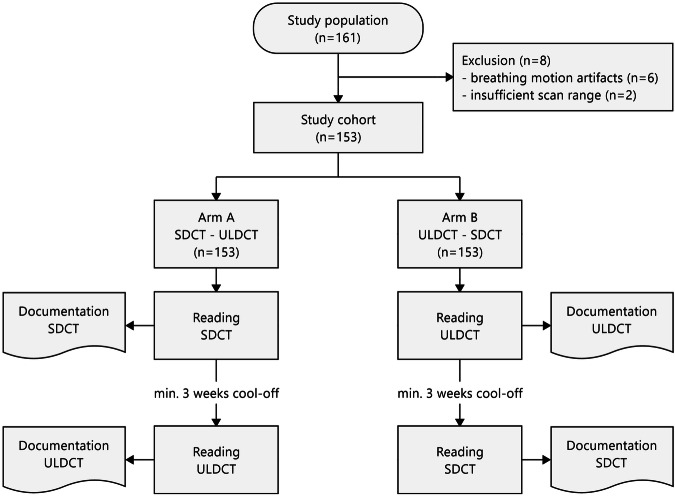
Study flow diagram. In this trial, we conducted a same-day, intra-patient comparison of ULDCT and SDCT of the chest with regard to agreement and accuracy for post-COVID-19 lung changes

In this study, 161 patients were recruited from May 2020 to January 2021. Eight patients (5.0%) were excluded from the image analysis due to breathing motion artifacts during one of the two CT scans (*n* = 6) or insufficient scan range (*n* = 2). Thus, the final cohort consisted of 153 patients (Fig. 1).

Of the 153 analyzed patients, 74 (48.4%) were women, and the mean age was 47.4 ± 15.3 years. The median time between the diagnosis of COVID-19 and the CT scan was 93 days (67; 131). Patient characteristics are detailed in Table 2.

**Table 2:** Patient characteristics

Patient characteristics	
Sex	74 women (48.4%); 79 men (51.6%)
Age (mean ± SD)	47.4 years (± 15.3)
Height (median [IQR])	174 cm (167; 180)
Weight (median [IQR])	75 kg [68; 90]
BMI (median [IQR])	25.7 kg/m² (23.0; 29.3)
Days after COVID-19 diagnosis (median [IQR]; mean ± SD)	93 days (67; 131); 105 ± 51.6

*IQR* interquartile range

### CT findings

According to the reference standard, findings were consistent with post-COVID lung abnormalities in 45 (29.4%) patients. The most frequent finding was GGO (*n* = 45; 100.0%), followed by reticulations (20/45; 44.4%), parenchymal bands (17/45; 37.8%), and bronchial enlargements (14/45; 31.1%), as detailed in Table 3. The mean extent of post-COVID lung abnormalities, according to the reference standard, was 28.5% ± 3.3, most commonly affecting the lower lobes—right 42/45 (93.3%) and left 37/45 (82.2%). A peripheral distribution was present in 29/45 patients (64.4%).

**Table 3:** Prevalence of CT findings according to the reference standard

Prevalence of CT findings	
Post-COVID lung abnormalities	29.4% (45/153)
Ground-glass opacifications	29.4% (45/153)
Reticulations	13.1% (20/153)
Parenchymal bands	11.1% (17/153)
Bronchial enlargements	9.2% (14/153)
Consolidations	6.5% (10/153)
Honeycombing	0% (0/153)
Crazy paving	0% (0/153)

Prevalence among patients with post-COVID lung abnormalities	
Ground-glass opacifications	100.0% (45/45)
Reticulations	44.4% (20/45)
Parenchymal bands	37.8% (17/45)
Bronchial enlargements	31.1% (14/45)
Consolidations	22.2% (10/45)
Honeycombing	0% (0/45)
Crazy paving	0% (0/45)

### Diagnostic performance of ULDCT

**Fig. 2:**
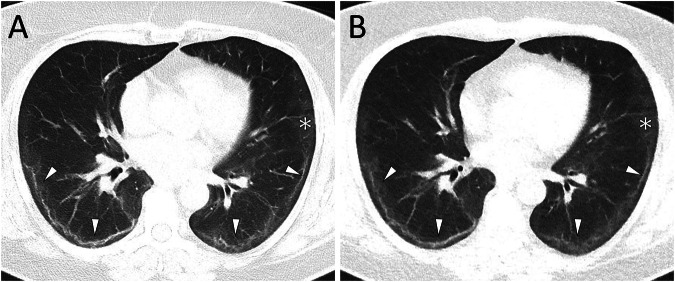
This female patient (110 days after COVID-19 diagnosis) showed extensive subpleural parenchymal bands in both lungs (white arrowheads) as well as ground glass opacifications (asterisks), clearly distinguishable with SDCT (**A**, DLP 148.9 mGy*cm) and ULDCT (**B**, DLP 14.9 mGy*cm) at the same slice thickness and reconstruction interval. Images are shown in the standard lung window of W/L 585/1230

**Fig. 3:**
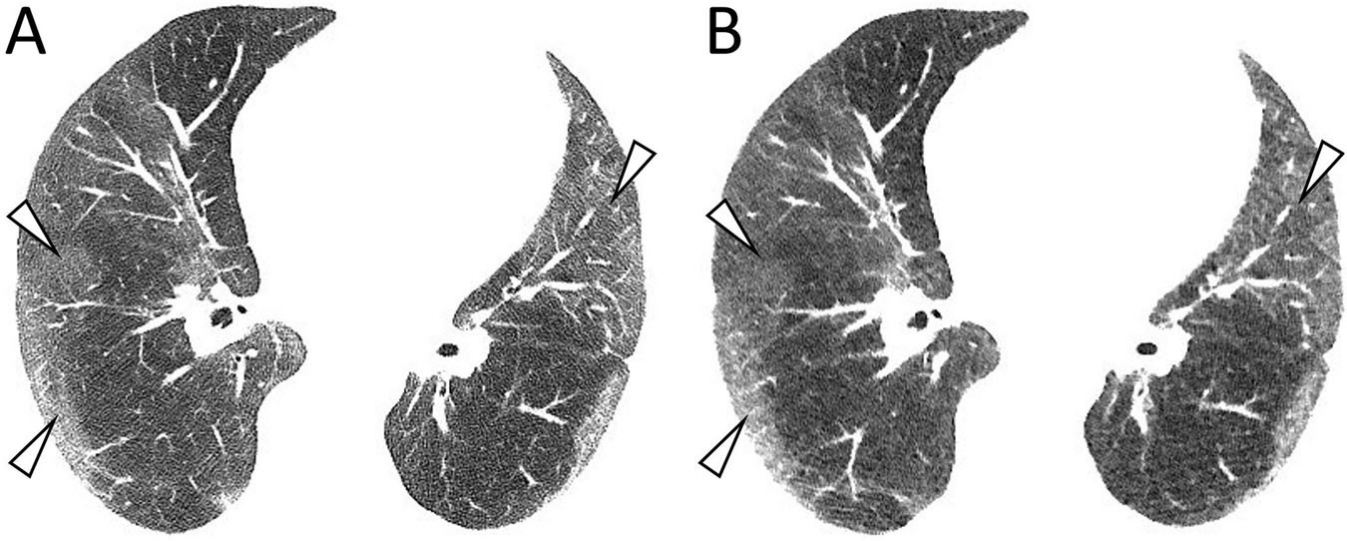
This obese male patient with a BMI of 38.2 (93 days after COVID-19 diagnosis) showed extensive bilateral ground glass opacifications with primarily peripheral distribution (white arrowheads), clearly visible with SDCT (**A**, DLP 300.9 mGy*cm) and ULDCT (**B**, DLP 30.9 mGy*cm) at the same slice thickness, reconstruction interval (3/2 mm). Images are shown in harder-than-standard windowing of W/L 800/500

**Fig. 4:**
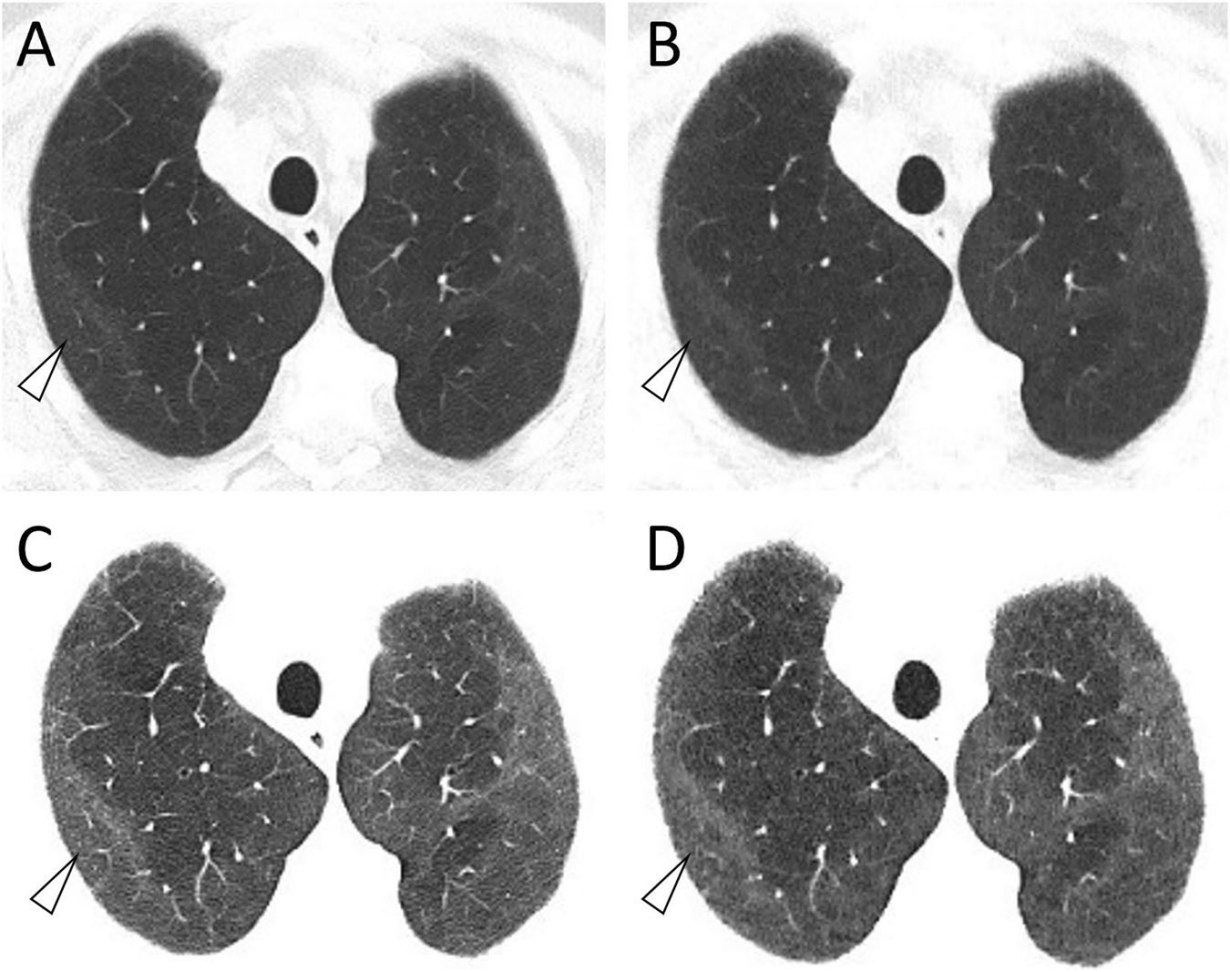
This male patient (130 days after COVID-19 diagnosis) showed discreet, but extensive ground glass opacifications (GGO) in both lungs (white arrowheads). These can be clearly identified with both SDCT (**A,** **C**; slice thickness 3 mm; DLP 109.5 mGy*cm) and ULDCT (**B,** **D**; slice thickness 3 mm; DLP 10.0 mGy*cm), especially with harder windowing of W/L 800/500 (**C,** **D**) in contrast to a standard lung window of W/L 585/1230 (**A,** **B**)

**Fig. 5:**
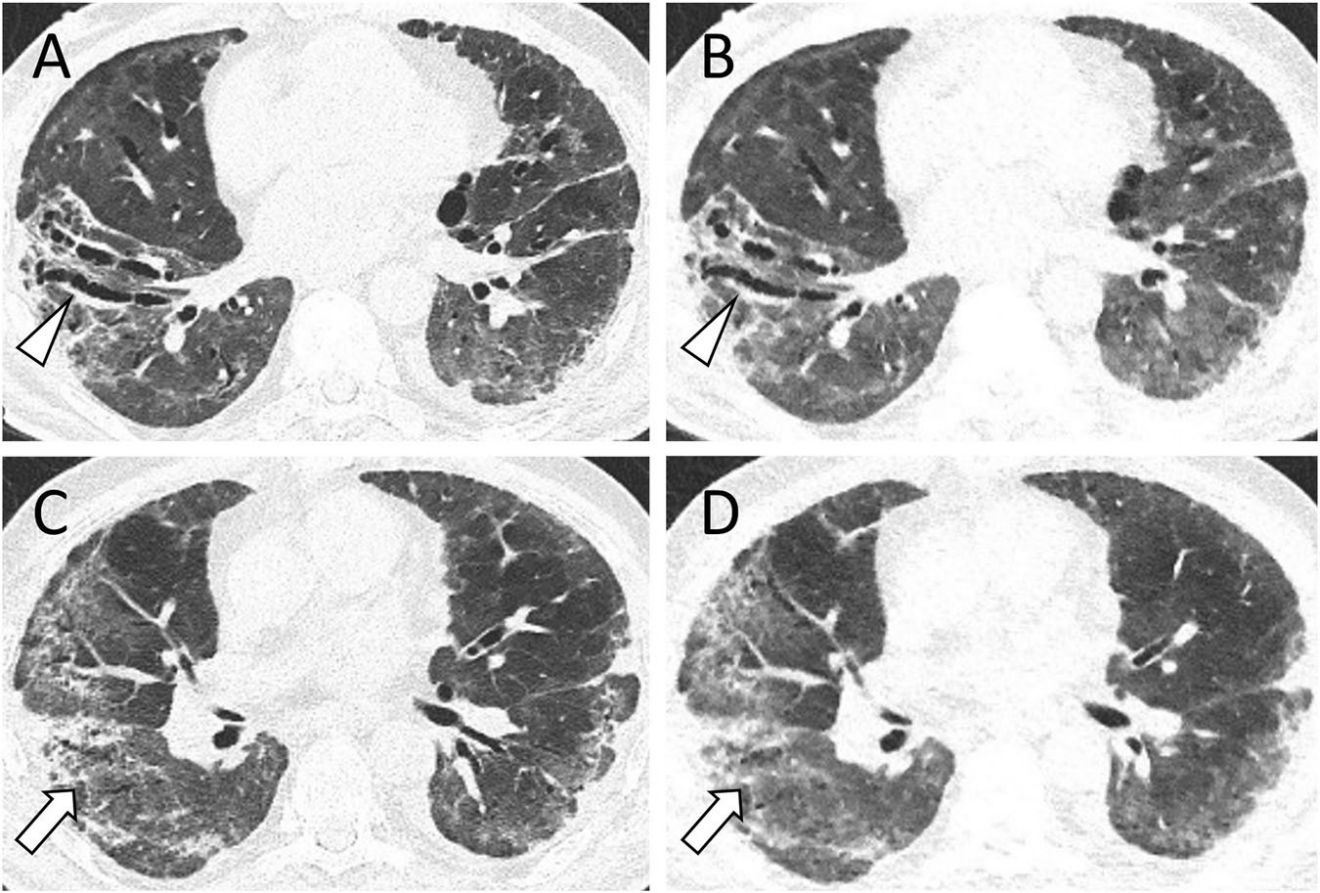
This male patient (80 days after COVID-19 diagnosis) showed multiple bronchial enlargements (**A**, **B**; white arrowheads), clearly visible with both SDCT (**A**, DLP 139.8 mGy*cm) and ULDCT (**B**, DLP 11.5 mGy*cm). In addition, extensive reticulations were present (**C**, **D**; white arrows), which could be better appreciated with SDCT, but are still visible with ULDCT (**D**). Images were reconstructed with the same slice thickness and reconstruction interval (1/0.8 mm) and are shown in the standard lung window of W/L 585/1230

Overall mean intrareader agreement of ULDCT with SDCT for all readers regarding the presence of post-COVID lung abnormalities was 93.3%. For the most frequent findings, agreement of ULDCT with SDCT was 93.8% for GGO, 90.0% for parenchymal bands, 94.0% for reticulations, 92.8% for bronchial enlargement, and 97.5% for consolidations. The intrareader agreement of ULDCT did not significantly differ between patients with an examination more than 12 weeks after the onset of COVID-19 and those with shorter intervals (*p* = 0.269). Results are provided in Table 4 and imaging examples of both ULDCTs and corresponding SDCTs are displayed in Figs. 2–5.

**Table 4:** Sensitivity, specificity, and accuracy of ULDCT and intrareader agreement of ULDCT with SDCT for the overall cohort together with results for examinations more than 12 weeks after onset of COVID-19 in parenthesis

Finding	Sensitivity ULDCT	Specificity ULDCT	Accuracy ULDCT	Agreement ULDCT with SDCT
Post-COVID lung abnormalities	87.2% (85.9%)	94.9% (94.2%)	92.6% (92.1%)	93.3% (91.8%)
Ground-glass opacifications	88.0% (87.0%)	93.2% (93.7%)	91.7% (91.8%)	93.8% (93.5%)
Reticulations	47.4% (42.5%)	95.5% (95.7%)	89.5% (89.9%)	94.0% (94.0%)
Parenchymal bands	82.1% (81.3%)	92.0% (90.6%)	90.7% (89.4%)	90.0% (87.8%)
Bronchial enlargements	60.7% (68.8%)	95.9% (95.9%)	92.6% (92.7%)	92.8% (92.7%)
Consolidations	80.0% (75.0%)	98.1% (98.3%)	96.9% (96.7%)	97.5% (97.3%)

Note: No patients displayed honeycombing or crazy paving

Analysis of the influence of the patients’ body mass index (BMI) and sex, as well as the individual reader, on the agreement of ULDCT with SDCT regarding the presence of post-COVID lung abnormalities, showed the reader (*p* = 0.001) to be the only significant factor. Neither a BMI of ≥ 30 (*p* = 0.214) nor sex (*p* = 0.323) significantly influenced agreement.

The sensitivity and specificity of ULDCT for the detection of findings that were regarded as consistent with post-COVID lung abnormalities were 87.2 and 94.9%, respectively, with an accuracy of 92.6%. For specific findings, the sensitivity and specificity for GGO were 88.0 and 93.2% (accuracy of 91.7%), for parenchymal bands 82.1 and 92.0% (accuracy 90.7%), for reticulations 47.4 and 95.5% (accuracy 89.5%), for bronchial enlargement 60.7 and 95.9% (accuracy 92.6%), and for consolidations 80.0 and 98.1% (accuracy 96.9%). The sensitivity, specificity, and accuracy of ULDCT did not significantly differ between patients with an examination more than 12 weeks after the onset of COVID-19 and those with shorter intervals (sensitivity *p* = 0.589; specificity *p* = 0.658; accuracy *p* = 0.781). Results are provided in Table 4. The detected mean extent of affected lung volume did not significantly differ between ULDCT and SDCT, with 30.5% (± 3.4) and 28.5% (± 3.3), respectively (*p* = 0.085).

### Radiation dose

**Fig. 6:**
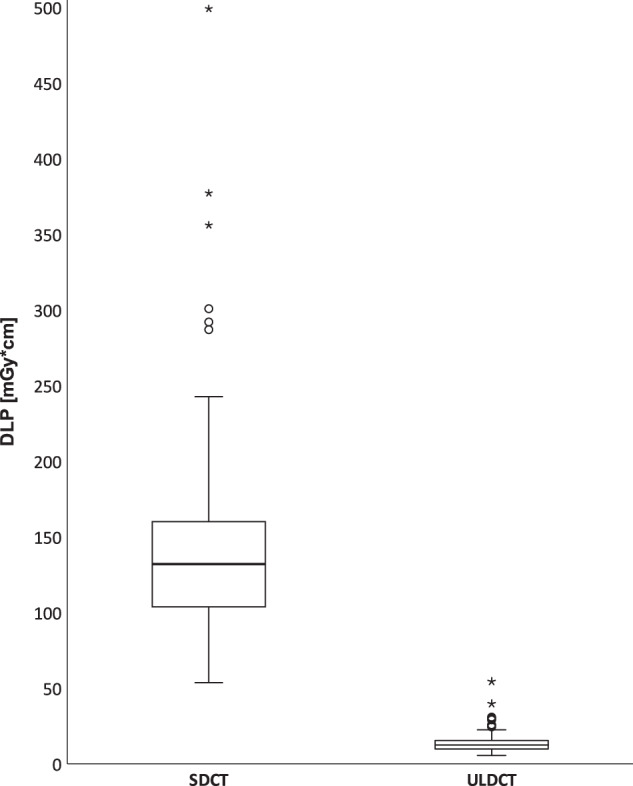
Radiation dose of standard-of-care-dose CTs (SDCT) and ultra-low-dose CTs (ULDCT) in this study as dose length product (DLP, mGy*cm). Circles (°) represent mild outliers (> 3rd quartile + 1.5 × IQR or < 1st quartile – 1.5 × IQR), asterisk (*) represent extreme outliers (> 3rd quartile + 3 × IQR or < 1st quartile – 3 × IQR)

For SDCTs, the median total DLP was 132.1 mGy*cm (103.9; 160.2), ranging from 53.7 to 499.2 mGy*cm. This corresponds to a median ED of 2.24 mSv (1.75; 2.70), ranging from 0.91 to 8.49 mSv. ULDCTs required a median total DLP of 12.6 mGy*cm (9.9; 15.5) with a range of 5.6 to 54.5 mGy*cm. This corresponds to a median ED of 0.21 mSv (0.17; 0.26), ranging from 0.10 to 0.93 mSv. At less than one-tenth, the radiation dose of ULDCTs was significantly lower than that of SDCTs (*p* < 0.001). A visualization is provided in Fig. 6.

### Image quality

Subjective image quality was significantly lower for ULDCT with a median of 3.0 (IQR 2.0; 3.0) ranging from 2 to 4 than for SDCT with a median of 5.0 (4.0; 5.0), ranging from 2 to 5 respectively (*p* < 0.001). However, all ULDCT and SDCT scans were rated as diagnostic image quality (image quality ≥ 2).

### Interreader agreement

Regarding the presence of post-COVID lung abnormalities, interreader agreement was substantial, with a Fleiss Kappa of 0.802 for SDCT (95%CI: 0.737, 0.867) and 0.745 for ULDCT (CI: 0.680, 0.809). Fleiss Kappa for specific findings were similar for both dose levels, showing the best results for GGO, with 0.738 for SDCT (CI: 0.673, 0.802) and 0.739 for ULDCT (CI: 0.674, 0.803). Detailed results are provided in Table 5.

**Table 5:** Inter-reader agreement for ULDCT and SDCT, Fleiss Kappa

Finding	ULDCT (ĸ)	SDCT (ĸ)
Post-COVID lung abnormalities	0.745	0.802
Ground-glass opacifications	0.739	0.738
Reticulations	0.286	0.428
Parenchymal bands	0.541	0.573
Bronchial enlargements	0.452	0.603
Consolidations	0.558	0.671

No patients displayed honeycombing or crazy paving

The intraclass correlation coefficient for inter-reader agreement regarding the extent of affected lung volume was good for ULDCT, with 0.769 (CI: 0.621, 0.873) and SDCT, with 0.712 (CI: 0.473, 0.852).

## Discussion

In this prospective study of a cohort of 153 patients with post-COVID conditions, ULDCT of the chest was compared to our institutional SDCT performed during the same examination for the detection of post-COVID lung abnormalities. The results show excellent intra-reader agreement of ULDCT with SDCT and high overall diagnostic accuracy regarding the detection of CT-morphological post-COVID lung abnormalities at less than one-tenth the radiation dose. Therefore, ULDCT of the chest may provide a viable radiation-saving alternative to SDCT in the large patient cohort of patients with post-COVID conditions, including many young individuals who will potentially require multiple follow-up CTs over time.

Intrareader agreement of ULDCT with SDCT regarding the presence of overall post-COVID lung abnormalities was excellent, at 93.3%, ranging from 87.6 to 96.1% for individual readers. For specific patterns of post-COVID lung abnormalities, agreement ranged from 92.8% for bronchial enlargement to 98.4% for crazy paving. In addition, ULDCT offered very good sensitivity (87.2%), and an even higher specificity (94.9%) regarding the general presence of post-COVID lung abnormalities. However, its sensitivity for the specific imaging patterns showed a broad variance ranging from 47.4 to 88.0%, while specificity ranged only from 92.0 to 100%. ULDCT offered the highest sensitivity for GGO and parenchymal bands, followed by consolidations, and low sensitivity for reticulations and bronchial enlargement. In addition, inter-reader agreement was best for GGO, consolidations and parenchymal bands and worst for reticulations and bronchial enlargement, with results similar to the literature [44–47]. Notably, inter-reader agreement for reticulations and bronchial enlargement was lower for ULDCT than for SDCT, indicating a dose dependency in this regard. However, intra-reader agreement of ULDCT with SDCT for all findings was excellent, including reticulations and bronchial enlargement ranging from 90.0 to 97.5%. Therefore, the low to moderate sensitivity of ULDCT for the findings of reticulations and bronchial enlargements in this study is likely mainly attributable to the lower inter-reader agreement, partially in combination with the lower radiation dose of ULDCT compared to SDCT. Importantly, the lower subjective image quality of ULDCT did not appear to impair intrareader agreement for the presence of post-COVID lung abnormalities, although the ULDCT scans were acquired at less than 10% of the radiation dose of SDCTs, corresponding to approximately twice the radiation dose of a standard CXR examination in two views [31–33].

Previous studies have investigated the diagnostic performance of SDCT and ULDCT in acute COVID-19 pneumonia patients. Agostini et al reported the feasibility and comparable diagnostic image quality of a ULDCT scan protocol compared to standard-dose high-resolution dual-energy CT in a cohort of ten acutely hospitalized COVID-19 patients at a median DLP of 19.5 mGy*cm (ED 0.28 mSv) and of 226.2 mGy*cm (ED 3.3 mSv), respectively [25]. Argentieri et al conducted a retrospective study comparing ULDCT to chest radiographs in symptomatic hospitalized COVID-19 patients and found substantially more lung injuries, as well as better inter-reader agreement with ULDCT [27]. Garg et al included 60 COVID-19 patients who were examined with standard-dose CT (SDCT; ED 4.93 mSv) and ULDCT (ED 0.26 mSv) [48]. In addition to objective and subjective image quality, the two dose levels were assessed for a range of lung abnormality patterns characteristic of acute COVID-19 pneumonia, such as GGO, consolidation, or septal thickening. ULDCT showed excellent results compared to SDCT as the reference standard for the CT severity score and measurements of diagnostic accuracy for the detection of typical lung abnormalities. However, to date, no studies have investigated the diagnostic accuracy of ULDCT in a cohort of post-COVID patients. Therefore, this study complements previous assessments by extending the evidence regarding the applicability of ULDCT to the large patient cohort of post-COVID patients. Since the prevalence of lung abnormalities on imaging has been shown to be linked to the proportion of patients with dyspnea, these may need multiple follow-up chest CT examinations over time and include many young individuals [49, 50]. Lifetime radiation exposure in this population is of particular importance and should be minimized in accordance with the ALARA principle.

The imaging characteristics of this post-COVID patient cohort are in line with similar cohorts reported in the literature, as 29.4% of patients presented with findings consistent with post-COVID lung abnormalities. The most frequent findings were GGOs, reticulations, parenchymal bands, and bronchial enlargements [17, 18, 51].

This study has several limitations. First, it was conducted at a single center. Second, our cohort consisted of unselected patients with post-COVID conditions referred to our department for routine follow-up chest CT. Thus, our sample may be skewed toward milder cases, including those with little or no morphological post-COVID lung abnormalities. However, this likely represents the patient population in clinical practice, and, given the high sensitivity, the applied ULDCT protocol detected even subtle lung abnormalities. Third, patients with pre-existing lung conditions were not excluded from this study and may have partially been interpreted as being consistent with post-COVID lung abnormalities, independent of the radiation dose level. Fourth, this study did not perform histopathological correlation, hence false-positive findings by SDCT and missed lung abnormalities in ULDCT and SDCT scans cannot be ruled out. Fifth, this study aimed to analyze the radiological detectability of post-COVID lung abnormalities with ULDCT in comparison to SDCT and did not attempt clinical correlation.

In conclusion, ULDCT of the chest offers excellent intrareader agreement with our institutional SDCT and high accuracy for the detection of CT-morphological post-COVID lung abnormalities at less than one-tenth the radiation dose of SDCT, corresponding to only twice the ED of a standard chest radiograph in two views (0.1 mSv). Therefore, ULDCT of the chest may provide a favorable radiation-saving alternative to SDCT in a large patient cohort of patients with post-COVID conditions.

### Data sharing statement

The anonymized participant data on which this study is based will be made available upon request by email to the corresponding author by qualified researchers after publication. No imaging data exceeding the images provided in this manuscript can be made available. Proposals will be approved by the authors on the basis of scientific merit and the absence of competing interests after the signing of a data access agreement and confidentiality agreement.

## Abbreviations

As: Ampere-seconds
BMI: Body mass index
CDC: Centers for Disease Control and Prevention
COVID-19: Coronavirus disease 2019
CT: Computed tomography
DLP: Dose length product
ED: Effective dose
GGO: Ground glass opacities
kVp: Kilovoltage peak
RI: Reconstruction interval
SDCT: Standard-of-care-dose CT
ST: Section thickness
ULDCT: Ultra-low-dose computed tomography
